# Supplementary material for: Istaroxime treatment ameliorates calcium dysregulation in a zebrafish model of phospholamban R14del cardiomyopathy
Source: Nat Commun. 2021 Dec 9;12:7151. doi: 10.1038/s41467-021-27461-8 (PMC8660846; doi:10.1038/s41467-021-27461-8)
Supplement: Supplementary file 1 — Supplementary Information [file 41467_2021_27461_MOESM1_ESM.pdf]

Istaroxime treatment ameliorates calcium dysregulation in a zebrafish model for phospholamban R14del cardiomyopathy

Kamel S.M.[1,*], van Opbergen C.J.M.[2,*], Koopman C.D.[1,2,*], Verkerk A.O.[3,4], Boukens, B.J.D[3,4], de Jonge B.[3], Onderwater Y. L.[1], van Alebeek E.[1],Chocron S.[1], Polidoro Pontalti C. [2], Weuring W.J[5], Vos M.A.[2], de Boer T.P.[2], van Veen T.A.B.[2,#], Bakkers J.[1,2,6,#]

SUPPLEMENTARY INFORMATION

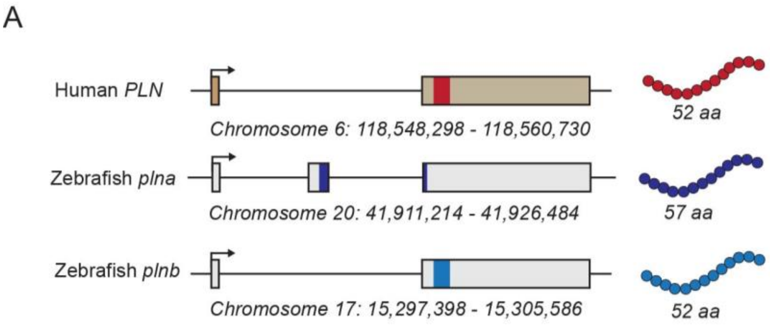

B

Species	Protein sequence	Length
H. sapiens	MEKVQYLTRSAIR**RAST**IEMPQQARAKLQNLFINFCLILICLLLICIIVML	52 aa
D. rerio plna	MEKVQHMTRAAIR**RAST**MEVPQQAKQNMQELFVNFCLILICLLLIYIIVLLISFHCM	57 aa
D. rerio plnb	MERVQHMTRSAIR**RAS**NIEVNPQTKRNLQDLIINFSLILICLLLIYIIVLLM	52 aa

Ia Ib II

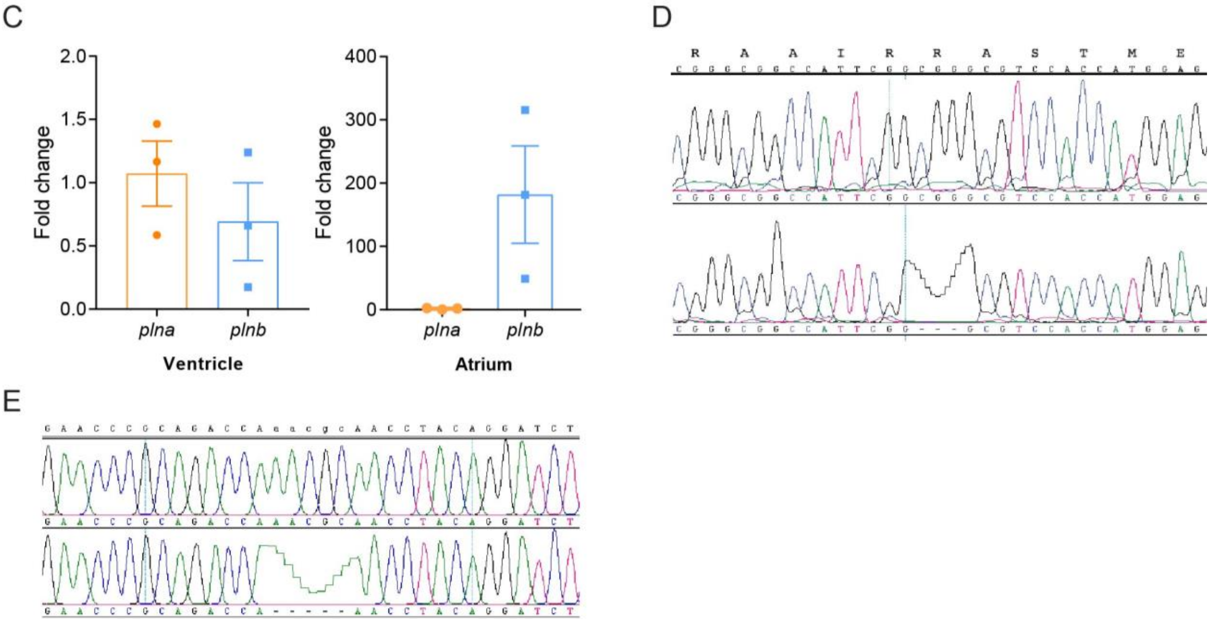

Figure S1. Description of zebrafish *plna* and *plnb*. A) In contrast to humans, zebrafish carry two *pln* genes. One gene is located on chromosome 20 (*plna*) and one on chromosome 17 (*plnb*). The R14del mutation was introduced to *plna*. B) Alignment of the human (*H. sapiens*) and zebrafish (*D. rerio*) protein sequences. Differences in amino acid sequence are indicated in red. The R14 and phosphorylation sites are indicated in bold. The different protein domains are indicated by Ia (cytosolic), Ib (linker), and II (transmembrane). C) Quantitative PCR plot shows the expression levels of *plna* (in orange) and *plnb* (in blue) in ventricle (n=3), atrium (n=3) and whole heart (n=3) of wild-type fish (mean±SEM, unpaired Students t-test). D) Sequencing peaks of *plna* showing the R14del mutation from a wild-type and a *plna* R14del mutant fish. In the R14del fish, the Arginine on amino acid location 14 has evidently been deleted. E) Sequencing peaks of *plnb* showing a 5bp deletion in a mutant compared to a wild-type. aa: amino acids.

A PLN R14del sgRNA gene specific region blasted: GGCACGGGCGGCCATTCGGC

Potential off-target	Off-target sequence	Primers off-target analysis
Nrp1a (intron 10)	sgRNA: GGCACGGGCGGCCATTCGGC<br>Nrp1a: GGCACGGGCGGCCATAATGA	Fw: TGCTATGACCTCTGCGTGAC<br>Rv: TGGGGTTTTGGATGTTATGC
Thrb (intron 2)	sgRNA: GGCACGGGCGGCCATTCGGC<br>Thrb: TGGGCGGGCGGCCATTCGGC	Fw: GAAACGAAGCCCAGTGGAAG<br>Rv: ACGACCGAGTTCGAGTCCAG
Toak2a (intron 3)	sgRNA: GGCACGGGCGGCCATTCGGC<br>Toak2a: GGCACGGGCGGCCATGCTCT	Fw: GTACAGGTGTGGGGTGCTCT<br>Rv: GAGCCATGGTGTCCAGAACT

B

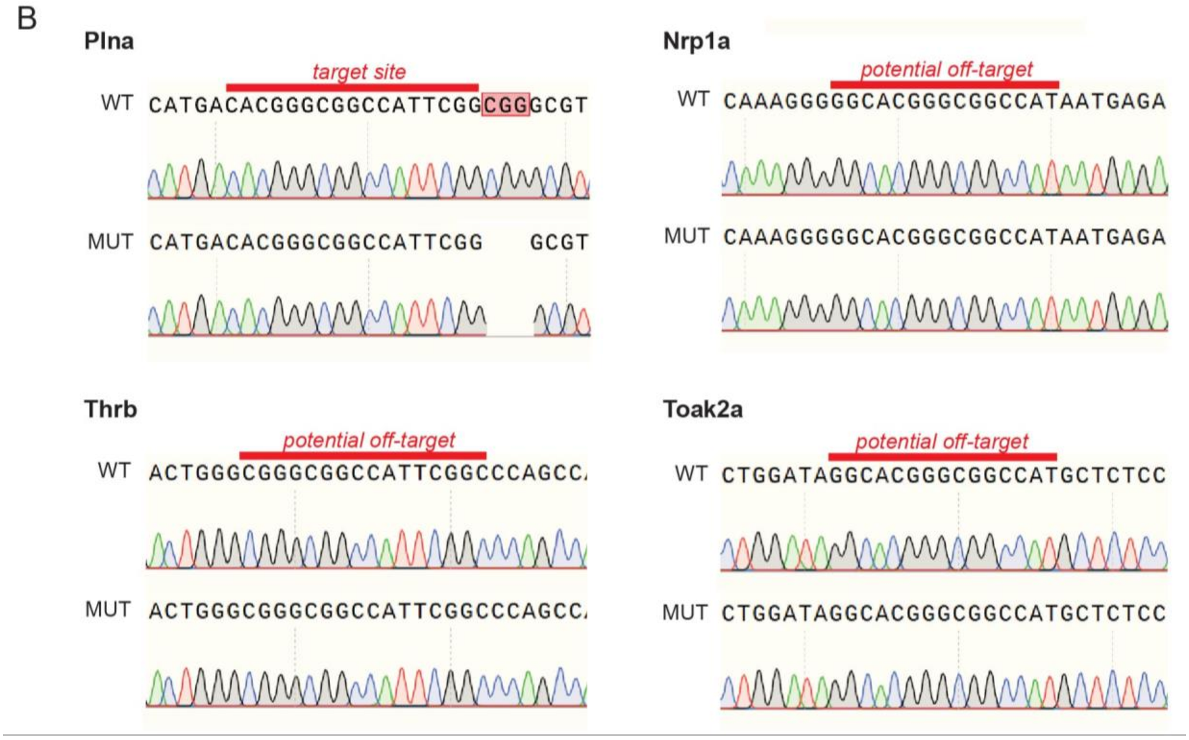

Figure S2. Analysis for possible off-target mutations. A) A Blastn against zebrafish GRCz11 with the sgRNA sequence that was used to target the *plna* gene resulted in three genomic regions (besides plna) with a significant overlap with the sgRNA sequence (highlighted in yellow). Note that all these regions are located in intronic regions. Primers were designed to check for possible off-target mutations in these regions. B) Sanger sequencing traces of the amplified region of the candidate off-target regions in wild-type and plna R14del mutants. As expected, only the *plna* region was mutated while the other regions showed a wild type sequence in the *plna* R14del mutants (Wild-type n=10, plna R14del n=20). Fw: Forward primer, Rv: Reverse primer, WT: Wild-type, MUT: *plna* R14del mutants.

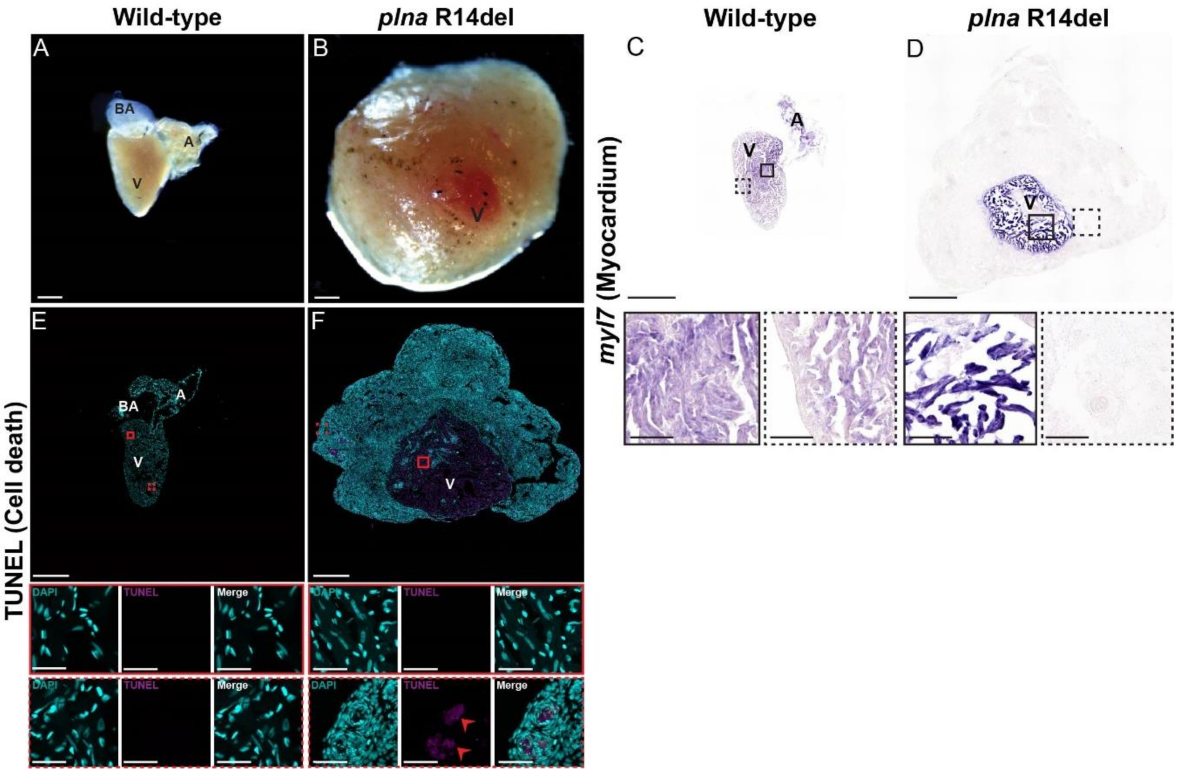

Figure S3. Fibrofatty replacement and cell death in adult *plna* R14del hearts. A-B) Bright field images of isolated wild-type and *plna* R14del mutant zebrafish hearts (10 months). C-D) *in situ* hybridization using *myl7* to mark the myocardium in wild-type and *plna* R14del mutant zebrafish hearts (10 months). E-F) TUNEL fluorescent staining to indicate apoptosis, where DAPI+ nuclei are shown in cyan and TUNEL+ nuclei in magenta. All stainings were performed on WT n=3, *plna* R14del n=3, two experimental replicates. Zoom-in images showing the expression of DAPI and TUNEL, staining of TUNEL in *plna* R14del is highlighted with red arrows. Images were taken at a magnification of 20x for whole heart and at a zoom of 63x for TUNEL staining. Scale bars are 200µm for whole heart tile scans and 20µm or 50 µm for zoom-in regions at a magnification of 40x and 63x, respectively. A: atrium, V: ventricle, BA: bulbus arteriosus.

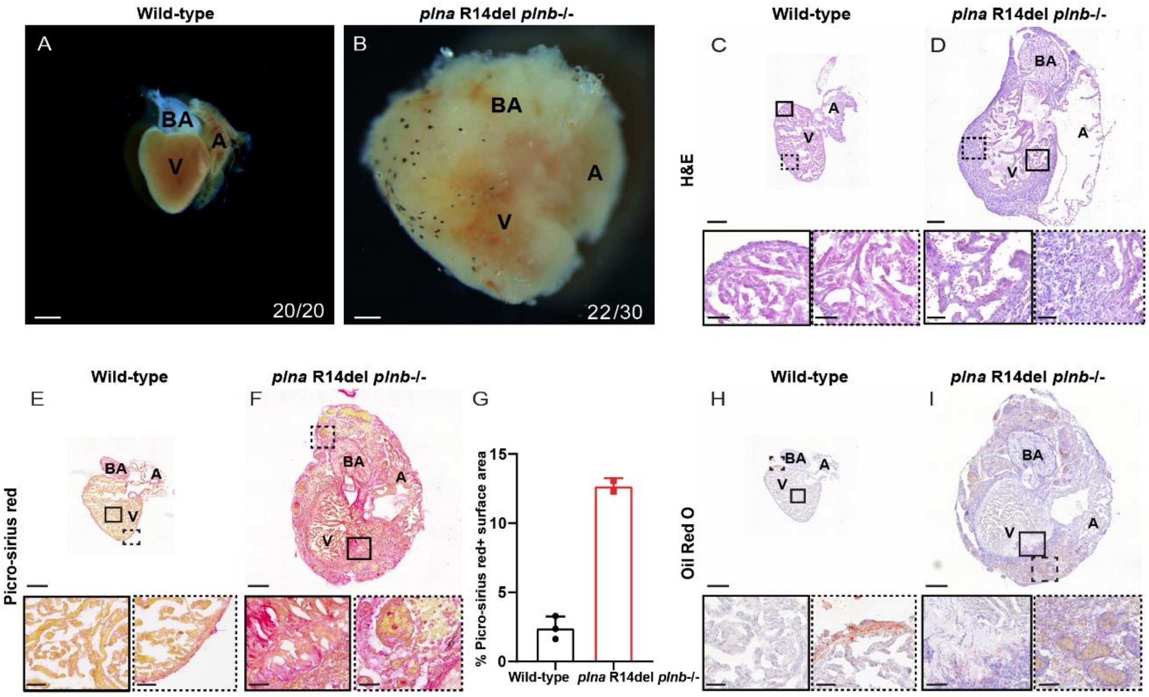

Figure S4. Structural remodeling of the adult *plna* R14del *plnb* -/- zebrafish heart. A-B) Bright field images of isolated adult zebrafish hearts, 1 years of age: wild-type fish, remodeled *plna* R14del *plnb*-/- mutant heart C-D) Haematoxylin and Eosin staining of the two conditions to identify nuclei, with zoom-in at indicated regions (WT n=3, *plna* R14del n=3, two experimental replicates). E-F) Picro-sirius red staining of collagen deposition for the two conditions, collagen fibers are shown as red staining. G) Quantification of Picro-sirius red staining in wildtype (in black) and *plna* R14del *plnb*-/- mutant heart (in red) (mean±SEM, WT n=3, *plna* R14del n=2, unpaired Students t-test) H-I) Oil Red O staining for fat/lipid on the hearts of the two conditions is shown (WT n=3, *plna* R14del n=3, two experimental replicates). Zoom-in of each indicated region is included. Images were taken at a magnification of 20x. Scale bars are 200µm for whole heart tile scans and 50µm for zoom-in regions. A: atrium, V: ventricle, BA: bulbus arteriosus.

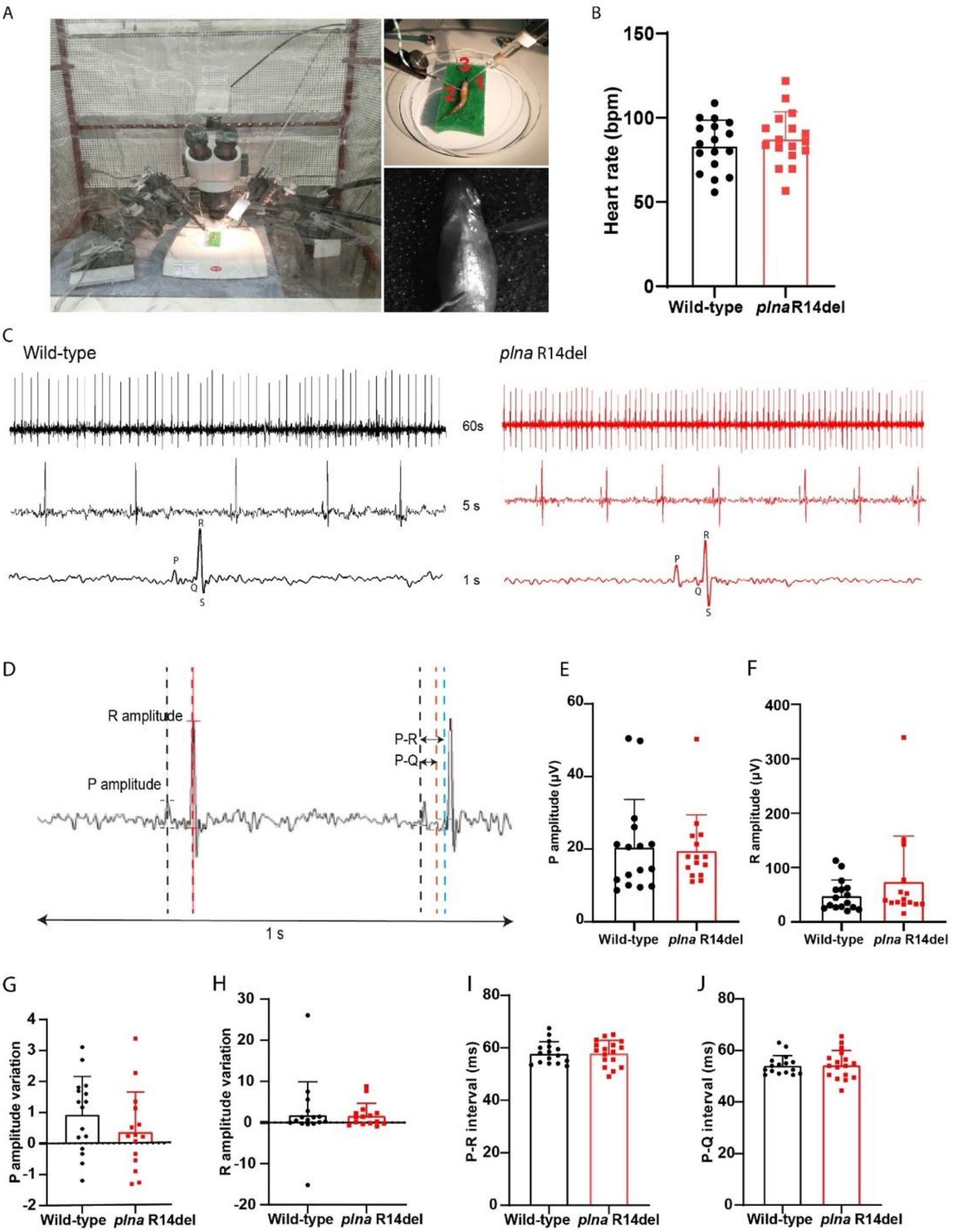

Figure S5. Electrocardiogram (ECG) recording of adult *plna* R14del zebrafish. A) The microscope set up for the ECG recordings and zoom in on the fish where 1 indicates grounding wire, 2 indicates reference electrode and 3 indicates the recording electrode. B) Heart rate measurements of both conditions. C) Sample recording for both conditions for 60s, 5s and 1s, displaying the P wave and the QRS complex. D) Illustration of the measurements conducted on the recordings where we show the P amplitude, R amplitude, PR interval and PQ interval. E) P amplitude quantification for both conditions. F) R amplitude quantification for both conditions. G-H) Variation in P and R amplitude of both conditions, respectively. I-J) P-R and P-Q intervals of each condition, respectively (mean±SEM, $p>0.05$, WT n=17, *plna* R14del n=18). Statistics were performed using unpaired Students t-test. Wildtype are highlighted in black and *plna* R14del in red. bpm: beats per minute, µV: micro volts, ms: miliseconds. Source data are provided as a Source Data file.

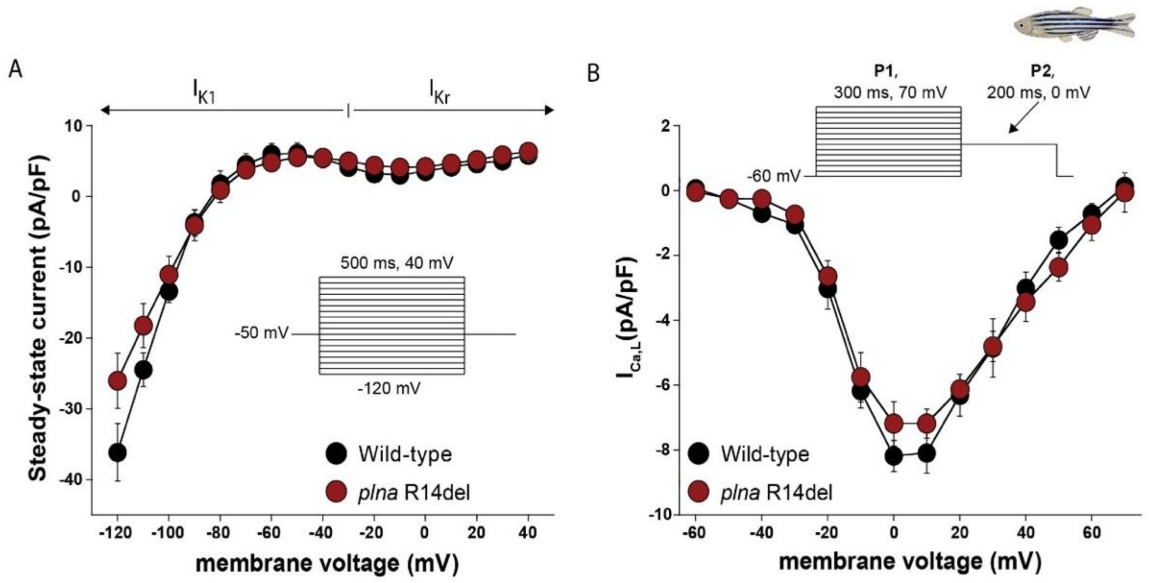

Figure S6. Effect of the *plna* R14del mutation on membrane currents in isolated cardiomyocytes. A) Current-voltage (I-V) relationships of I_{K1} and I_{Kr} in cardiomyocytes from wild-type (WT) and *plna* R14del mutant zebrafish (mean ± SEM, unpaired Students t-test, three experimental replicates). Inset, voltage clamp protocol used. B) I-V relationships of $I_{Ca,L}$ in WT and *plna* R14del zebrafish cardiomyocytes (mean±SEM, p>0.05, unpaired Students t-test, three experimental replicates). Inset, voltage clamp protocol used. Wildtype are highlighted in black and *plna* R14del in red. WT: wild type, ms: milliseconds, mV: millivolt, I_{K1}: inward rectifier potassium current, I_{Kr}: rapidly activated delayed rectifier potassium current, $I_{Ca,L}$: L-type calcium current. Source data are provided as a Source Data file.

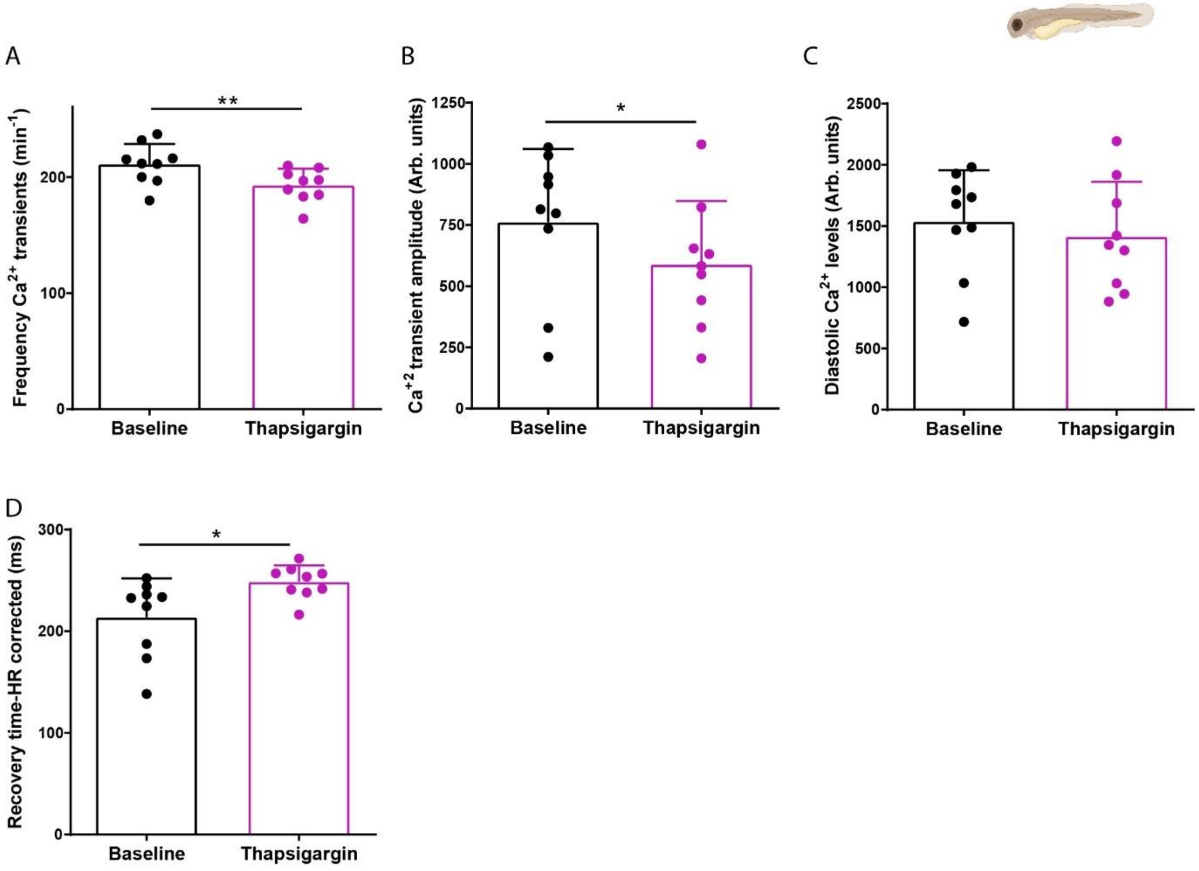

Figure S7. *In vivo* effect of Thapsigargin on calcium transient amplitude in GCaMP6f embryos. A-D) Effect of 100 μM thapsigargin on the frequency of calcium (Ca^{2+}) transient, Ca^{2+} transient amplitude, diastolic Ca^{2+} levels and recovery time in GCaMP6f zebrafish (mean ± SEM, (A) **p=0.0029, (B) * p=0.0271, (D) *p=0.0458, baseline n=9, thapsigargin n=9). Statistical analysis was performed using paired Students t-test. All measurements were performed in two experimental replicates. Baseline condition is highlighted in black and treated condition in magenta. min^{-1}: per minute, Arb. units: arbitrary units, ms: milliseconds. Source data are provided as a Source Data file.

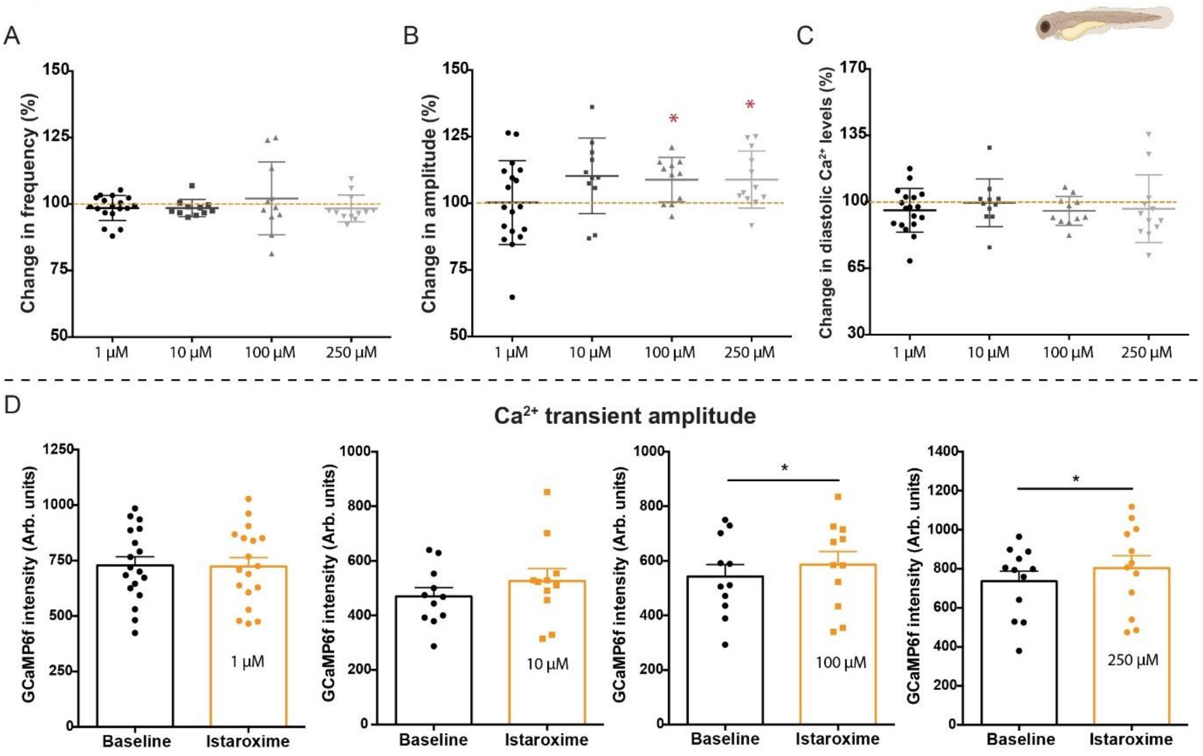

Figure S8. Dose response effect of istaroxime in GCaMP6f embryos. A-C) Effect of increasing concentrations of istaroxime (1-250 µM) on calcium (Ca^{2+}) transient parameters in GCaMP6f zebrafish. Values are presented as relative change (in %) from grouped baseline values of all concentrations. Parameters include A) the frequency of Ca^{2+} transients, B) the Ca^{2+} transient amplitude and C) diastolic Ca^{2+} levels (mean ± STDEV, *p=0.0111, baseline n=51, 1 µM n= 17, 10 µM n= 11, 100 µM n= 11, 250 µM n= 12). D) The effect of different istaroxime concentrations (1-250 µM) on the Ca^{2+} transient amplitude in GCaMP6f zebrafish. Bar graphs represent baseline values (in black) and values after incubation with istaroxime (in orange) (mean ± SEM, *p=0.0129, 1 µM n= 18, 10 µM n= 11, 100 µM n= 11, 250 µM n= 12) Statistical analysis was performed using paired Students t-test. All measurements were performed in two experimental replicates. Arb. units: arbitrary units. Source data are provided as a Source Data file.

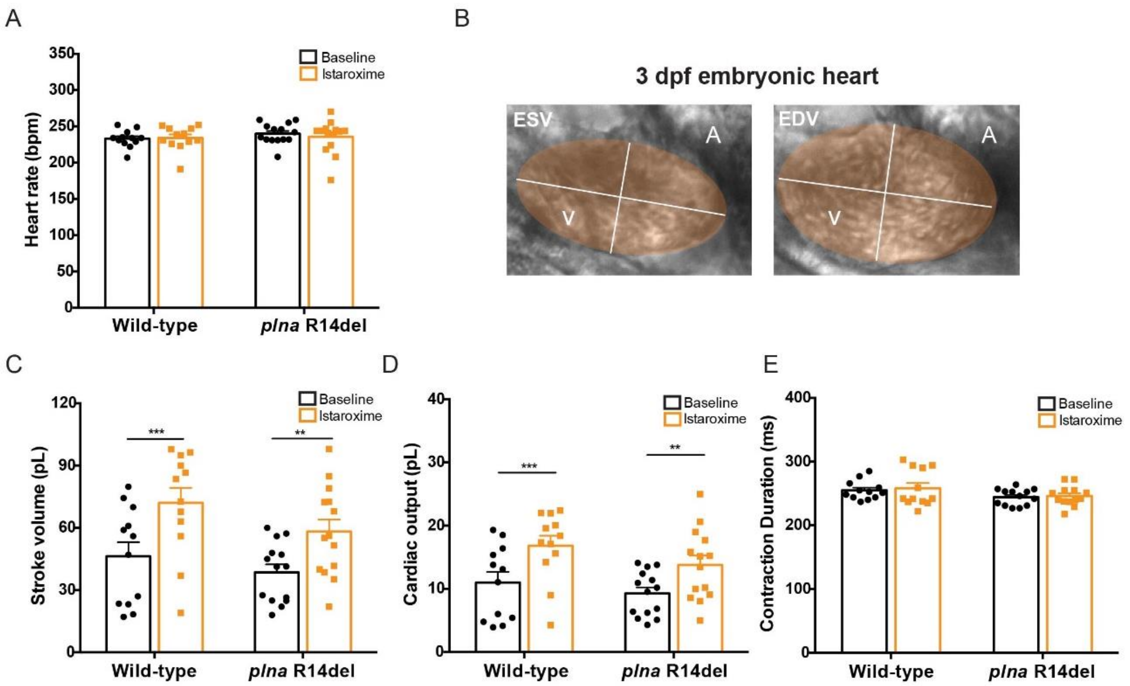

Figure S9. Effect of istaroxime on cardiac contractility parameters in wild-type and *plna* R14del mutants. A-E) Cardiac contractility parameters examined in wild-type (WT) and *plna* R14del mutant embryos at baseline and after incubation with 100 µM istaroxime. Heart rate is shown in (A). To measure hemodynamic parameters in high speed imaging recordings, we used ImageJ to fit an ellipse over the ventricle in every recording, both at end-systole (left) and end-diastole (right) (B). This allowed the extraction of contractility measurements, such as stroke volume (C) (mean ± SEM, ** p=0.0016, *** p=0.0002, WT baseline n=12, WT istaroxime n=12, *plna* baseline n=14, *plna* istaroxime n=14), cardiac output (D) (mean ± SEM, ** p=0.0011, *** p=0.0001, WT baseline n=12, WT istaroxime n=12, *plna* baseline n=14, *plna* istaroxime n=14) and contractile cycle length contraction duration (E). Statistical analysis was performed using paired Students t-test. All measurements were performed in three experimental replicates. Wildtype are highlighted in black and *plna* R14del in orange. bpm: beats per minute, ESV: end systolic volume, EDV: end diastolic volume, pL: Pico litre, ms: milliseconds. Source data are provided as a Source Data file.

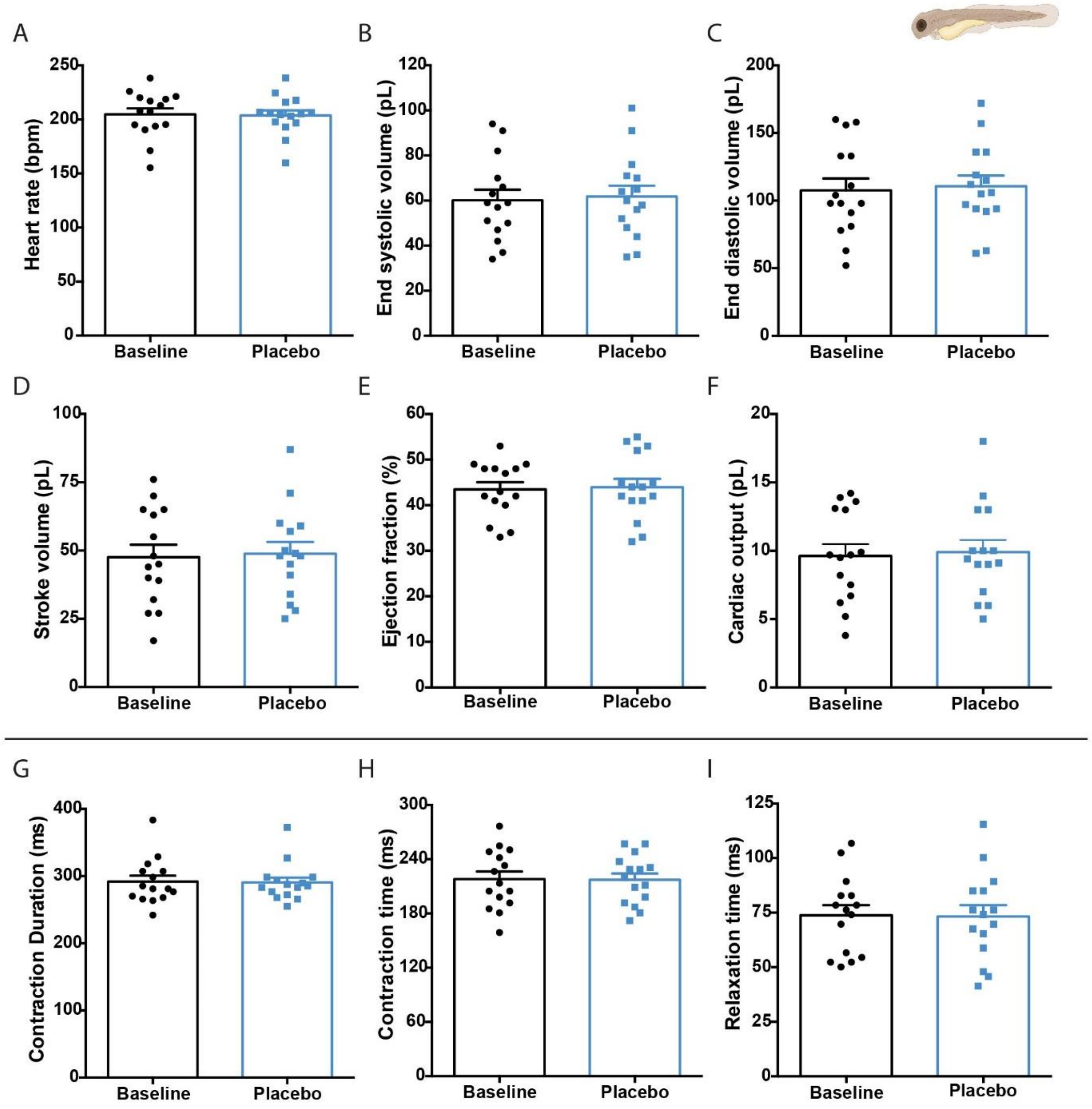

Figure S10. Effect of placebo treatment on cardiac contractility parameters in wild-type embryos. A-I) Cardiac contractility parameters examined in wild-type (WT) embryos at baseline, and 30 minutes after replacing the incubation medium (E3 water + MS222) with the exact similar medium (mean ± SEM, baseline n=15, placebo n=15, all statistics were performed using paired Students t-test), including heart rate (A), end systolic volume (B), end diastolic volume (C), stroke volume (D), ejection fraction (E), cardiac output (F), contractile cycle length contraction duration (G), contractile cycle length contraction time (H) and contractile cycle length relaxation time (I). All measurements were performed in two experimental replicates. Baseline condition is highlighted in black and placebo in blue. bpm: beats per minute, pL: Pico litre, ms: milliseconds. Source data are provided as a Source Data file.

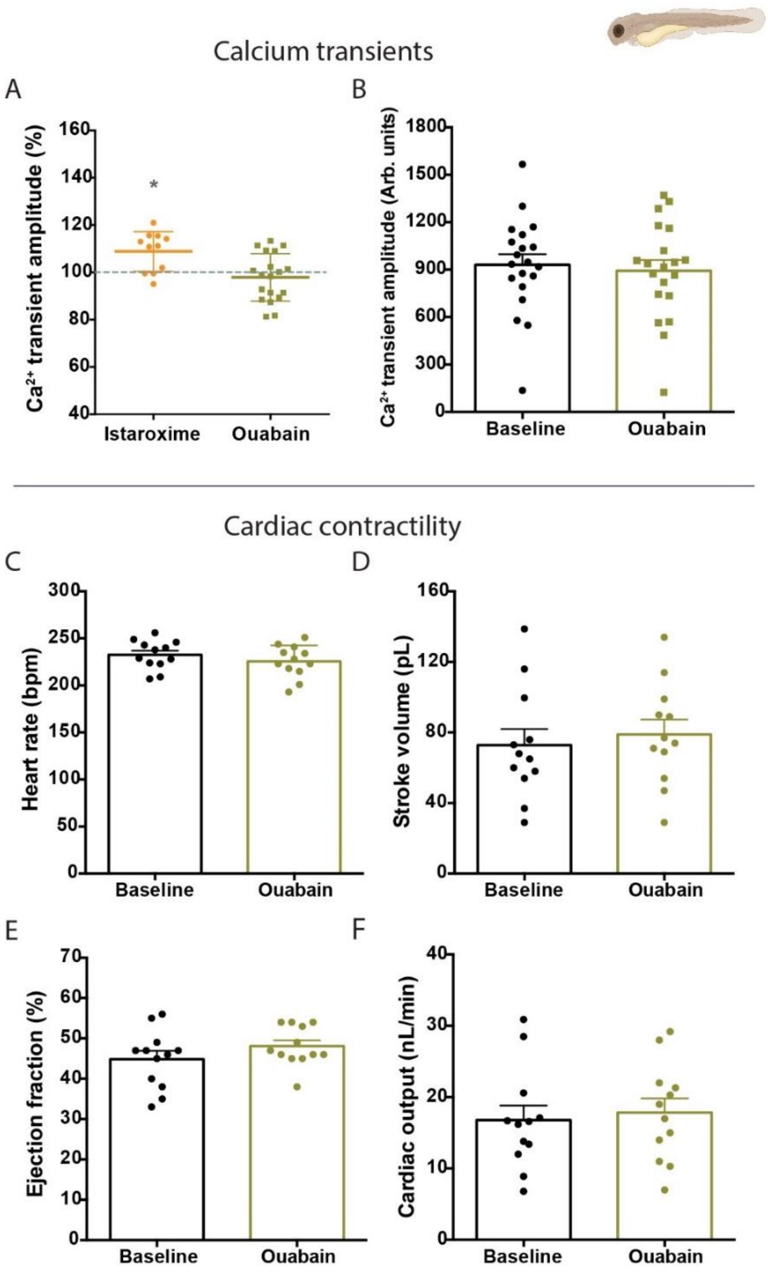

Figure S11. In vivo effect of ouabain on calcium transient amplitude and cardiac contractility in GCaMP6f embryos. A) Effect of 100 µM istaroxime and 100 µM ouabain on the calcium (Ca^{2+}) transient amplitude in GCaMP6f zebrafish. Values are presented as relative change (in %) from baseline values (mean ± SEM, * $p \leq 0.05$, baseline n=35, istaroxime n=15, ouabain n=20, paired Students t-test). B) The Ca^{2+} transient amplitude at baseline and after incubation with 100 µM ouabain (mean ± SEM, n=20, paired Students t-test). All measurements were performed in two experimental replicates. C-F) Cardiac contractility parameters examined in GCaMP6f embryonic zebrafish at baseline and after incubation with 100 µM ouabain (mean ± SEM, baseline n=12, ouabain n=12, all statistical analysis were performed using paired Students t-test), including heart rate (C), stroke volume (D), ejection fraction (E) and cardiac output (F). All measurements were performed in two experimental replicates. Baseline condition is highlighted in black and Ouabain in green. bpm: beats per minute, Arb. units: arbitrary units, pL: Pico litre, nL/min: nanolitre per minute. Source data are provided as a Source Data file.

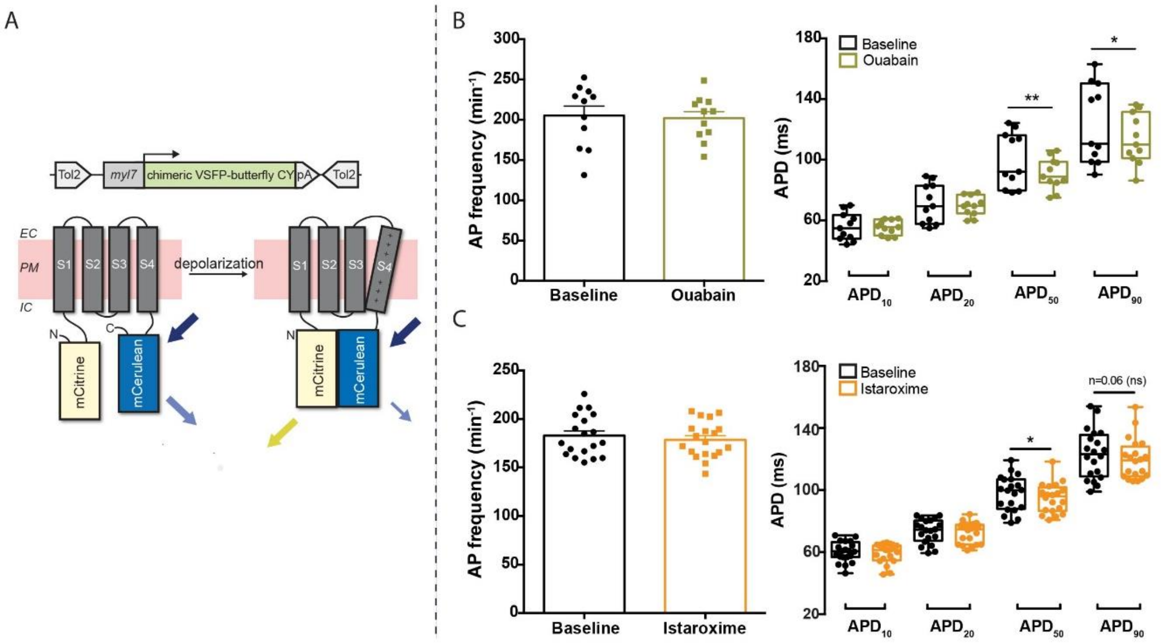

Figure S12. In vivo effect of istaroxime and ouabain on action potential parameters in VSFP-butterfly CY embryos. A) DNA construct and concept of the sensing mechanism of chimeric voltage sensitive fluorescent protein (VSFP)-butterfly CY. Chimeric VSFP-butterfly CY was placed under control of the *myl7* promoter to restrict its expression to the heart. The senor consists of a voltage sensitive domain with transmembrane segments S1-S4, sandwiched between a fluorescence resonance energy transfer (FRET) pair of the fluorescent proteins mCitrine and mCerulean. Movement of S4 upon membrane depolarization translates into a change of FRET efficiency. B) Frequency of action potentials and APD parameters at baseline and after incubation with 100 µM ouabain (mean ± SEM, * p=0.0161, ** p=0.0068, n=11). All measurements were performed in two experimental replicates. C) Frequency of action potentials and APD parameters at baseline and after incubation with 100 µM istaroxime (mean ± SEM, * p=0027, n=19). All measurements were performed in three experimental replicates. Baseline condition is highlighted in black, istaroxime in orange and Ouabain in green. The values in the box and whiskers plots are indicated by dots for individual measurements, where the highest value on the upper whisker is the maxima (100th percentile), lowest value on the lower whisker is the minima (0th percentile), the bounds of the box indicates the 25th- 75th percentile and the line in the box plot indicates the median (50th percentile). Statistical analysis was performed using paired Students t-test min^{-1}: per minute, ms: milliseconds, APD_{10}: AP duration at 10% of repolarization, APD_{20}: AP duration at 20% of repolarization, APD_{50}: AP duration at 50% of repolarization, APD_{90}: AP duration at 90% of repolarization. Source data are provided as a Source Data file.

Table S1. List of primers used to generate in situ hybridization probes and for qPCR reaction. ZF: zebrafish, ISH: in situ hybridization, F: forward primer, R: reverse primer.

Primer name	Primer sequence
ZF_ISH_*grn1*_ F	TCCCGGTGGAGACTGTAGAC
ZF_ISH_*grn1*_ R	AATGACGGTGCATTTTGACA
ZF_ISH_*postnb*_ F	AGAGGTTCTGGACAGGCTCA
ZF_ISH_*postnb*_ R	AAGGCACCATTTTTCACCAG
ZF_qPCR_*plna*_F	TCTCCACTGCCATCTCTCCT
ZF_qPCR_*plna*_R	ACGAAGAGCTCCTGCATGTT
ZF_qPCR_*plnb*_F	ACCAGCCTCATCATCTCCAC
ZF_qPCR_*plnb*_R	ATCCTGTAGGTTGCGTTTGG
ZF_qPCR_*ef1 α* _F	CTTCTCAGGCTGACTGTGC
ZF_qPCR_*ef1 α* _R	CCGCTAGCATTACCCTCC

Table S2. Echocardiographic measurements of 6 and 10 months old wild-type (WT) and *plna* R14del zebrafish. Values are mean ± SEM. For VOT diameter: WT (** p= 0.0093) and PLN (*** p= 0.00026), for VOT surface: WT (** p=0.0072) and PLN(*** p= 0.00037), for VOT PV: WT (* p=0.0165) and PLN (** p=0.0013), for stroke volume: WT (** p=0.0018) and PLN (**** p=0.00009),for cardiac output: WT (** p=0.0013) and PLN (*** p= 0.0004) and for VOT PV variation: PLN (* p= 0.01954). All statistical analysis was performed using one way ANOVA. VOT; ventricular outflow tract, PV; peak velocity, bpm; beats per minute, mm; millimeters, s; second, pL; Pico litre, mL/min, milliliter per minute.

	Echocardiographic parameters							
	n	Heart rate (bpm)	VOT diameter (mm)	VOT surface (mm^2)	VOT PV (mm/s)	Stroke volume (pL)	Cardiac output (mL/min)	VOT PV variation (mm/s)
**Wildtype 6 Months**	10	92.39 ± 4.08	0.379 ± 0.014	0.203 ± 0.028	100.79 ± 11.50	2.88 ± 0.476	0.236 ± 0.049	4.43 ± 0.46
**Plna R14del 6 Months**	10	99.75 ± 5.29	0.368 ± 0.021	0.219 ± 0.025	101.64 ± 10.33	2.65 ± 0.53	0.253 ± 0.051	5.78 ± 0.84
**Wildtype 10 Months**	21	98.97 ± 4.93	0.440 ± 0.009 **	0.306 ± 0.011 **	146.31 ± 6.57 *	5.74 ± 0.41 **	0.554 ± 0.042 **	2.97 ± 0.332
**Plna R14del 10 Months**	21	99.93 ± 3.92	0.446 ± 0.008 ***	0.314 ± 0.010 ***	159.07 ± 10.20 **	5.99 ± 0.40 ****	0.552 ± 0.038 ***	6.30 ± 0.85 *

***p 6 months vs. 10 months**

***p WT vs. *plna* R14del 10 months**
