# Supplementary material for: Istaroxime treatment ameliorates calcium dysregulation in a zebrafish model of phospholamban R14del cardiomyopathy
Source: Nat Commun. 2021 Dec 9;12:7151. doi: 10.1038/s41467-021-27461-8 (PMC8660846; doi:10.1038/s41467-021-27461-8)
Supplement: Supplementary file 3 — Reporting Summary [file 41467_2021_27461_MOESM3_ESM.pdf]

nature portfolio

Corresponding author(s): Jeroen Bakkers

Last updated by author(s): Sep 20, 2021

Reporting Summary

Nature Portfolio wishes to improve the reproducibility of the work that we publish. This form provides structure for consistency and transparency in reporting. For further information on Nature Portfolio policies, see our Editorial Policies and the Editorial Policy Checklist.

Statistics

For all statistical analyses, confirm that the following items are present in the figure legend, table legend, main text, or Methods section.

n/a	Confirmed	
☐	☒	The exact sample size (n) for each experimental group/condition, given as a discrete number and unit of measurement
☐	☒	A statement on whether measurements were taken from distinct samples or whether the same sample was measured repeatedly
☐	☒	The statistical test(s) used AND whether they are one- or two-sided<br>*Only common tests should be described solely by name; describe more complex techniques in the Methods section.*
☐	☒	A description of all covariates tested
☐	☒	A description of any assumptions or corrections, such as tests of normality and adjustment for multiple comparisons
☐	☒	A full description of the statistical parameters including central tendency (e.g. means) or other basic estimates (e.g. regression coefficient) AND variation (e.g. standard deviation) or associated estimates of uncertainty (e.g. confidence intervals)
☐	☒	For null hypothesis testing, the test statistic (e.g. F, t, r) with confidence intervals, effect sizes, degrees of freedom and P value noted<br>*Give P values as exact values whenever suitable.*
☒	☐	For Bayesian analysis, information on the choice of priors and Markov chain Monte Carlo settings
☒	☐	For hierarchical and complex designs, identification of the appropriate level for tests and full reporting of outcomes
☒	☐	Estimates of effect sizes (e.g. Cohen's d, Pearson's r), indicating how they were calculated

Our web collection on statistics for biologists contains articles on many of the points above.

Software and code

Policy information about availability of computer code

Data collection	Vevo 2100 Imaging System (VisualSonics Inc.), Scope 04.04.27 (custom-made software), Hokawo 2.9 Imaging Software (Hamamatsu Photonics), Micro-Manager 1.4.17 (Image software), Bio-Rad CFX Maestro 1.1 (Bio rad), LASX 3.4.2.18368 (Leica Microsystems), Labscribe NI (iworx).
Data analysis	Vevo 2100 analytical software (VisualSonics Inc.), MacDQ 10.7.1(custom-made software), ImageJ 1.52p (NIH), MATLAB R2015b (Mathworks), Imaris image analysis software (version 9.3.1), Microsoft Excel 2013 (Microsoft), GraphPad Prism V7 for Windows and V6 for Mac OS X (GraphPad software) and SigmaStat 3.5 software. We used cutom-made codes for MATLAB, these are publicly available on: [https://gitlab.com/OVeth/sarah/-/tree/master].

For manuscripts utilizing custom algorithms or software that are central to the research but not yet described in published literature, software must be made available to editors and reviewers. We strongly encourage code deposition in a community repository (e.g. GitHub). See the Nature Portfolio guidelines for submitting code & software for further information.

Data

Policy information about availability of data

All manuscripts must include a data availability statement. This statement should provide the following information, where applicable:

- Accession codes, unique identifiers, or web links for publicly available datasets
- A description of any restrictions on data availability
- For clinical datasets or third party data, please ensure that the statement adheres to our policy

The authors declare that all data supporting the findings of this study are available within the paper and its supplementary information. Source data for Figure 3, 4, 5, 6 and 7, and supplementary Figs. 1, 5, 6, 7, 8, 9, 10, 11 and 12 are provided with the paper. Due to the large sizes of the unprocessed raw images, these will be

available from the corresponding author upon reasonable request.

Field-specific reporting

Please select the one below that is the best fit for your research. If you are not sure, read the appropriate sections before making your selection.

[x] Life sciences   [ ] Behavioural & social sciences   [ ] Ecological, evolutionary & environmental sciences

For a reference copy of the document with all sections, see nature.com/documents/nr-reporting-summary-flat.pdf

Life sciences study design

All studies must disclose on these points even when the disclosure is negative.

Sample size	Sample size was determined based on previous studies from our group for embryonic and adult zebrafish (PMID: 33246633 and 26748692).
Data exclusions	No data were excluded from the analysis
Replication	The experimental finding presented presented in this study were reliably produced, with experimental replicate of 2-3.
Randomization	All analyzed zebrafish samples were chosen randomly
Blinding	Data collection on larvae were recorded and analyzed blindly, as genotyping of larvae was performed after the analysis. Adult zebrafish were genotyped and separated to wildtypes and mutants, analysis of adult zebrafish experiments were performed blindly.

Reporting for specific materials, systems and methods

We require information from authors about some types of materials, experimental systems and methods used in many studies. Here, indicate whether each material, system or method listed is relevant to your study. If you are not sure if a list item applies to your research, read the appropriate section before selecting a response.

Materials & experimental systems

n/a	Involved in the study	
[x]	[ ]	Antibodies
[x]	[ ]	Eukaryotic cell lines
[x]	[ ]	Palaeontology and archaeology
[ ]	[x]	Animals and other organisms
[x]	[ ]	Human research participants
[x]	[ ]	Clinical data
[x]	[ ]	Dual use research of concern

Methods

n/a	Involved in the study	
[x]	[ ]	ChIP-seq
[x]	[ ]	Flow cytometry
[x]	[ ]	MRI-based neuroimaging

Animals and other organisms

Policy information about studies involving animals; ARRIVE guidelines recommended for reporting animal research

Laboratory animals	Zebrafish (Danio rerio) were used in this study, where Tubingen longfin (TL) strain was used. Non-transgenic and transgenic animals were used, either wildtype or carrying Phospholamban R14 deletion (PLN R14del). PLN R14del fish were generated using CRISPR/Cas genome editing technology. This study used animals of mixed sex and age group between 6 months and 2 years depending on the experiment. Zebrafish larvae were used at 3 days post fertilization. Specific ages are provided in the methods section.
Wild animals	No wild animals were used
Field-collected samples	No field-collected samples were used
Ethics oversight	All experiments were conducted in accordance with the ethical guidelines and approved by the local ethics committee of the Royal Dutch Academy of Sciences (KNAW).

Note that full information on the approval of the study protocol must also be provided in the manuscript.
